# Comparison between hospital- and community-acquired septic shock in children: a single-center retrospective cohort study

**DOI:** 10.1007/s12519-022-00574-w

**Published:** 2022-06-23

**Authors:** Guo-Yun Su, Chao-Nan Fan, Bo-Liang Fang, Zheng-De Xie, Su-Yun Qian

**Affiliations:** 1 Pediatric Intensive Care Unit, National Center for Children’s Health, Beijing Children’s Hospital, Capital Medical University, No. 56 Nan-Li-Shi Road, Beijing, 100045 China; 2 Research Unit of Critical Infection in Children, Chinese Academy of Medical Sciences (DIFMS), 2019-I2M-5-026, Beijing, China; 3 Beijing Key Laboratory of Pediatric Respiratory Infection Diseases, Key Laboratory of Major Diseases in Children, Ministry of Education, National Clinical Research Center for Respiratory Diseases, Research Unit of Critical Infection in Children, Chinese Academy of Medical Sciences, 2019RU016, Laboratory of Infection and Virology, Beijing Pediatric Research Institute, Beijing Children’s Hospital, National Center for Children’s Health, Capital Medical University, Beijing, China

**Keywords:** 28-day mortality, Children, Community-acquired septic shock, Hospital-acquired septic shock, Septic shock

## Abstract

**Background:**

We explored the differences in baseline characteristics, pathogens, complications, outcomes, and risk factors between children with hospital-acquired septic shock (HASS) and community-acquired septic shock (CASS) in the pediatric intensive care unit (PICU).

**Methods:**

This retrospective study enrolled children with septic shock at the PICU of Beijing Children’s Hospital from January 1, 2016, to December 31, 2019. The patients were followed up until 28 days after shock or death and were divided into the HASS and CASS group. Logistic regression analysis was used to identify risk factors for mortality.

**Results:**

A total of 298 children were enrolled. Among them, 65.9% (*n* = 91) of HASS patients had hematologic/oncologic diseases, mainly with Gram-negative bacterial bloodstream infections (47.3%). Additionally, 67.7% (*n* = 207) of CASS patients had no obvious underlying disease, and most experienced Gram-positive bacterial infections (30.9%) of the respiratory or central nervous system. The 28-day mortality was 62.6% and 32.7% in the HASS and CASS groups, respectively (*P* < 0.001). Platelet [odds ratio (OR) = 0.996, 95% confidence interval (CI) = 0.992–1.000, *P* = 0.028], positive pathogen detection (OR = 3.557, 95% CI = 1.307–9.684, *P* = 0.013), and multiple organ dysfunction syndrome (OR = 10.953, 95% CI = 1.974–60.775, *P* = 0.006) were risk factors for 28-day mortality in HASS patients. Lactate (OR = 1.104, 95% CI = 1.022–1.192, *P* = 0.012) and mechanical ventilation (OR = 8.114, 95% CI = 1.806–36.465, *P* = 0.006) were risk factors for 28-day mortality in patients with CASS.

**Conclusions:**

The underlying diseases, pathogens, complications, prognosis, and mortality rates varied widely between the HASS and CASS groups. The predictors of 28-day mortality were different between HASS and CASS pediatric patients with septic shock.

## Introduction

Pediatric sepsis triggered by severe inflammatory responses to infections is the leading cause of mortality and morbidity worldwide, especially in children younger than 5 years old [1–3]. It occurs in up to 8% of pediatric intensive care unit (PICU) patients, causing 1 in 4 deaths with a mortality rate ranging from 7.8% to 40% [4, 5]. Children in developing countries have a significantly higher sepsis-related death rate compared to developed countries (31.7% vs. 19.3%) [6]. In China the mortality of Chinese children with severe sepsis and septic shock is as high as 34.6% [2]. Septic shock is a subset of sepsis, usually resulting in tissue necrosis, multiorgan failure, and death [7]. The precipitating factor of sepsis is an infection, which can be categorized as nosocomial or community-acquired infection according to the site of infection. The morbidity and mortality rates of sepsis caused by nosocomial infections are significantly different from those caused by community-acquired infections [8, 9]. Identifying the causes of these differences is essential to avoid the risk factors of death and to prevent and treat septic shock.

Published data on pediatric sepsis regarding the source of infection are very limited. Murni et al. [10] recently reported that among 2646 patients admitted to the PICU, 170 (6.4%) developed nosocomial sepsis, and 70 of these children died (fatality rate of 41%). In another multicenter prospective study of 136 children with sepsis in Spain, 22.8% (31/136) had nosocomial infections, of which 29.0% (9/31) died, and 7.6% (8/105) died of community sepsis [11]. In a prospective population-based cohort study with 444 episodes of blood culture-proven sepsis in 429 neonates, Giannoni et al. [12] reported that 20% of cases were early-onset sepsis, 62% cases were nosocomial late-onset sepsis, and 18% were community-acquired late-onset sepsis. They also showed that hospital-acquired sepsis occurred in infants of lower gestational age and was more frequently associated with comorbidities, wheras community-acquired sepsis was more common in term infants and male infants. Obtaining national or regional data is not an easy process, and data for such studies in Chinese patients is even scarcer. An analysis of a population-based database in China showed that among 21,191 hospitalized adult patients, 935 met the diagnosis of Sepsis-3 [7], among which 498 had severe sepsis or septic shock, and 62.1% of Sepsis-3 patients had community-acquired infections. The mortality rate of patients with severe sepsis or septic shock was 53.4% and that of patients with Sepsis-3 was 32.0% [13]. Studies on children and infants in developed countries, as well as on adults in developing countries, showed that there were differences in mortality and disease characteristics between nosocomial sepsis and community sepsis. Nevertheless, adequate data on nosocomial and community-acquired infections in children with septic shock in developing countries, especially China, have not yet been available.

This single-center retrospective study was designed to examine the differences in baseline characteristics, treatment, prognosis, outcomes, and risk factors of pediatric patients with hospital-acquired septic shock (HASS) and community-acquired septic shock (CASS), providing further guidance for the prevention and treatment of pediatric sepsis in clinical practice.

## Methods

### Study design and subjects

We conducted a 4-year, single-center, retrospective cohort study from January 1, 2016 to December 31, 2019. The study included eligible children from the PICU of Beijing Children’s Hospital. Patients who were 29 days to 18 years old were included, and all patients were diagnosed with pediatric septic shock according to the 2015 Chinese expert consensus diagnostic standard for children (septic shock was diagnosed if children who had sepsis had insufficient tissue perfusion and cardiovascular dysfunction) [14]: (1) hypotension (blood pressure below the 5th percentile for age or systolic blood pressure below the normal value of this age group by less than two standard deviations); (2) vasoactive drugs were needed to maintain blood pressure in normal range (dopamine > 5 μg/kg/min) or any dose of dobutamine, norepinephrine, or epinephrine; and (3) three manifestations of hypoperfusion among 1) peripheral artery pulsation was weak, heart rate and pulse increased rapidly, 2) pale or pale gray skin, wet and cold, marbled pattern, 3) the capillary refilling time (CRT) was prolonged (> 3 seconds) (except for the influence of ambient temperature), and the CRT could be normal during warm shock, 4) early irritability or malaise, apathy, late stage of confusion, even coma, convulsion, 5) after fluid resuscitation, the urine volume was still < 0.5 mL/kg/h for at least 2 hours, 6) arterial blood lactic acid > 2 mmol/L. Children with incomplete medical records and those who were lost to follow-up were excluded. The study protocol was reviewed and approved by the Ethics Committee of Beijing Children’s Hospital. Patient informed consent was waived.

### Groups

In this study the patients were divided into the HASS and CASS group according to whether the sepsis was due to nosocomial or community-acquired infections. The HASS group included patients who had infections 48 hours after admission and developed septic shock. The CASS group included patients who had infections on admission or within 48 hours after admission and had developed septic shock. Children who underwent hemodialysis or intravenous chemotherapy within 30 days or were hospitalized for more than 2 days in the past 90 days were classified as pediatric patients with healthcare-related infections and were also considered as HASS patients [15].

### Data collection

Clinical, demographic, diagnostic, antimicrobial, and etiological testing results; empirical antimicrobial therapy; and other treatment history, complications, and prognosis data of pediatric patients were collected from the clinical electronic medical record system (Jiahe System, Beijing). The 28-day survival after septic shock could not be identified from the medical record, and this information was obtained through telephone call follow-ups. Data were collected in the form of variable tables on a secured electronic database. The pediatric index of mortality 2 (PIM2) score was applied as a mortality prediction tool used in the PICU. The primary outcome of the present study was 28-day mortality. Secondary outcomes included in-hospital mortality, length of PICU stay, and length of hospital stay.

### Definitions

HASS and CASS were determined based on the definitions by the European Centers for Disease Prevention and Control [16]. According to expert recommendations of the 2012 European Committee for Antimicrobial Susceptibility Testing [16], multidrug-resistant (MDR) bacteria were defined as bacteria that obtained non-susceptibility to at least one agent in three or more antimicrobial categories. Extensively drug-resistant bacteria were defined as bacteria that were non-susceptible to at least one agent in all except two or fewer antimicrobial categories (i.e., bacterial isolates remain susceptible to only one or two categories). The definition of organ dysfunction in children was based on the recommendations of the 2005 International Pediatric Sepsis Conference [17, 18].

### Statistical analysis

All statistical analyses were performed using SPSS 23.0 (IBM Corp, Armonk, NY, USA). Kolmogorov–Smirnov test was used to verify the normality of the continuous data. Quantitative data with normal distribution were denoted by mean ± standard deviation, and quantitative data with a non-normal distribution were denoted by median and quartiles. Quantitative data with a normal distribution (or non-normal distribution) of the two groups were analyzed using the Student’s *t* test (or the Mann–Whitney *U* test as a nonparametric test). Categorical variables were presented as a count (percentage) and were analyzed using Pearson’s Chi-square test or continuous correction Chi-square test. To determine the risk factors for 28-day mortality of children with septic shock in the two groups, multivariable logistic regression analyses were conducted. The variables included in these analyses were the statistically significant variables in the univariable analyses and were tested for multicollinearity. The results of the multivariable logistic regression analyses were reported as adjusted odds ratio (OR) and 95% confidence interval (CI). Statistical significance was established at *P* < 0.05.

## Results

### General information

**Fig. 1:**
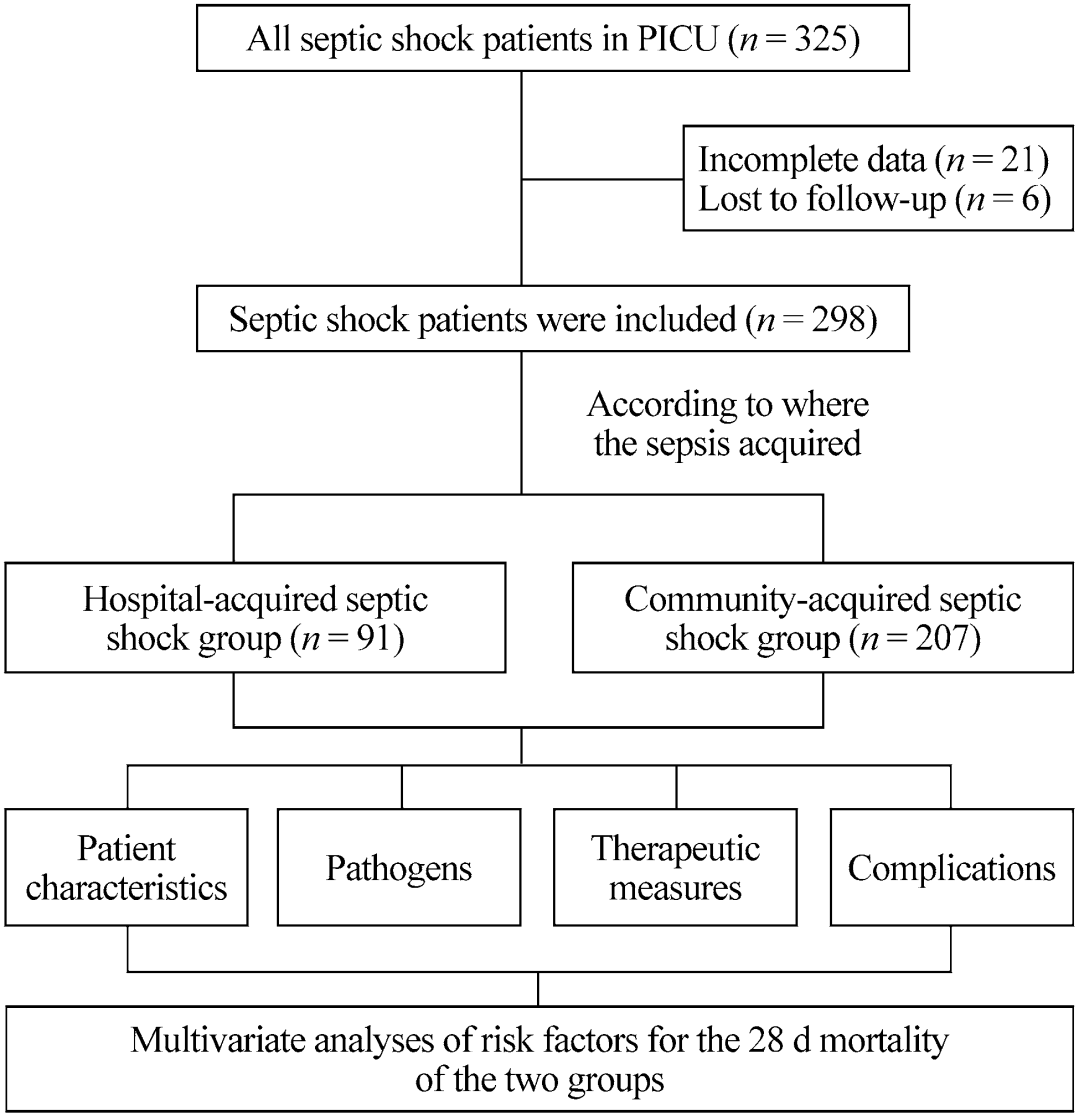
Flowchart of the study

In this study 325 children with septic shock were initially identified. Among them, 21 patients were excluded because their data were incomplete, and six patients were excluded because they were lost to follow-up. Finally, 298 patients were included in this study (Fig. 1). Among them, 91 (30.5%) patients were in the HASS group, and 207 (69.5%) were in the CASS group. In the HASS group, 37 (40.7%) patients were from the hematology department, 10 (11.0%) patients were from the PICU, five (5.5%) patients were from the surgery department, 11 (12.1%) patients were from other internal medicine departments, and 28 (30.8%) patients were from other hospitals. The median age of the HASS children was 5.2 (1.2–10.8) years, and 64.8% (59/91) were male. The PIM2 score at PICU admission was 9.6 (4.1–14.8) in the HASS group. In the CASS group, the median age of the children was 2.3 (0.6–8.2) years, 60.9% (126/207) of them were male, and the PIM2 score at the time of PICU admission was 8.5 (5.1–16.1) (Table 1). The characteristics of the pediatric patients are shown in Table 1.

**Table 1:** Demographics and clinical data of children with septic shock

Characteristics	HASS group (*n* = 91)	CASS group (*n* = 207)	*P*
Age (y), median (IQR)	5.2 (1.2, 10.8)	2.3 (0.6, 8.2)	0.002
Male, *n* (%)	59 (64.8)	126 (60.9)	0.516
PIM2 at PICU admission (%), median (IQR)	9.6 (4.1, 14.8)	8.5 (5.1, 16.1)	0.574
Underlying diseases, *n* (%)			
No obvious underlying disease	21 (23.1)	138 (67.7)	< 0.001
Hematologic/oncologic diseases	60 (65.9)	30 (14.5)	< 0.001
Immunodeficiency or autoimmune disease	3 (3.3)	4 (1.9)	0.763
Nervous system disease	3 (3.3)	12 (5.8)	0.534
Digestive tract disease	5 (5.5)	9 (4.3)	0.894
Prematurity	0 (0.0)	6 (2.9)	0.233
Congenital heart disease	1 (1.1)	6 (2.9)	0.596
Infection site, *n* (%)			
Digestive tract	26 (28.6)	44 (21.3)	0.170
Respiratory tract	6 (6.6)	52 (25.1)	< 0.001
Bloodstream	33 (36.3)	23 (11.1)	< 0.001
Central nervous system	9 (9.9)	46 (22.2)	0.011
Skin and soft tissue	7 (7.7)	17 (8.2)	0.879
Urinary system	0 (0.0)	3 (1.4)	0.600
Unclear site	10 (11.0)	22 (10.6)	0.926
Main complaints, *n* (%)			
Fever	65 (71.4)	169 (81.6)	0.048
Convulsions	9 (9.9)	47 (22.7)	0.009
Emesis	16 (17.6)	40 (19.3)	0.723
Disturbance of consciousness	10 (11.0)	50 (24.2)	0.009
Cough	8 (8.8)	44 (21.3)	0.009
Diarrhea	14 (15.4)	31 (15.0)	0.928
Listlessness	6 (6.6)	39 (18.8)	0.007
Rash	9 (9.9)	33 (15.9)	0.167
Abdominal pain	8 (8.8)	27 (13.0)	0.294
Shortness of breath	7 (7.7)	17 (8.2)	0.879
Crying	0 (0.0)	7 (3.4)	0.174
Vital signs at the time of shock			
Temperature (℃), median (IQR)	36.8 (36.5, 38.1)	37.4 (36.7, 38.1)	0.073
Abnormal RR, *n* (%)	70 (76.9)	167 (80.7)	0.460
Abnormal HR, *n* (%)	39 (42.9)	123 (59.4)	0.008
Abnormal SBP, *n* (%)	18 (19.8)	40 (19.3)	0.927
Blood biochemistry and hematologic indices, median (IQR)			
WBC (× 10^9^/L)	1.39 (0.15, 10.97)	8.45 (3.56, 17.32)	< 0.001
N (%)	63.1 (16.9, 82.2)	64.6 (44.8, 82.0)	0.134
PLT (× 10.^9^/L)	24 (9, 133)	147 (55, 257)	< 0.001
CRP (mg/L)	152.0 (72.8, 162.5)	81 (25, 160)	< 0.001
PCT (ng/mL)	17.5 (3.4, 61.0)	36.3 (6.0, 107.1)	0.081
PH	7.37 (7.27, 7.46)	7.35 (7.24, 7.42)	0.131
BE (mmol/L)	− 5.8 (− 11.8, − 0.43)	− 7.0 (− 11.2, − 4.0)	0.105
LAC (mmol/L)	2.7 (1.5, 6.3)	2.8 (1.5, 6.0)	0.969
ALB (g/L)	26.1 (23.0, 29.6)	26.1 (22.4, 30.9)	0.953
TBIL (μmol/L)	16.9 (8.9, 32.6)	10.9 (6.6, 18.8)	< 0.001
ALT (U/L)	32.3 (20.2, 74.3)	42.2 (21.1, 154.9)	0.092
APTT (s)	39.7 (32.0, 49.7)	43.0 (34.8, 54.8)	0.086
PT (s)	15.4 (12.9, 19.6)	15.8 (13.4, 19.8)	0.564
INR	1.37 (1.13, 1.74)	1.37 (1.17, 1.73)	0.580
ATIII (%)	63.0 (45.7, 87.0)	63.1 (46.5, 80.0)	0.892
d-Dimer (mg/L)	1.56 (0.57, 4.48)	2.45 (0.82, 6.02)	0.061
CR (μmol/L)	35.4 (21.8, 81.8)	45.1 (27.5, 82.2)	0.119

Quantitative data with normal distribution were represented as mean ± standard deviation, and quantitative data with non-normal distribution were represented as median and quartile. Classification variables were represented as count (percentage). *HASS* hospital-acquired septic shock, *CASS* community-acquired septic shock, *IQR* interquartile range, *PIM2* pediatric index of mortality 2, *PICU* pediatric intensive care unit, *RR* respiratory rate, *HR* heart rate, *SBP* systolic blood pressure, *WBC* white blood cell, *N* neutrophil ratio, *PLT* platelet, *CRP* C-reactive protein, *PCT* procalcitonin, *BE* base excess, *LAC* lactate, *ALB* albumin, *TBIL* total bilirubin, *ALT* alanine aminotransferase, *APTT* activated partial thromboplastin time, *PT* prothrombin time, *INR* international normalized ratio, *ATIII* antithrombin III, *CR* creatinine

### Infection sites and distribution of causative microorganisms

In this study the most common infection sites were the digestive tract, respiratory tract, bloodstream, and central nervous system. Bloodstream infection was dominant in the HASS group (36.3%) compared with the CASS group (11.1%). The CASS group had a higher number of patients with respiratory tract infections (25.1%) and central nervous system infections (22.2%) (Table 1).

The microbiological test results of patients are shown in Table 2. A statistical difference in pathogen distribution was observed. The patients in the CASS group had more Gram-positive bacterial infections than those in the HASS group (*P* < 0.001). Among Gram-positive pathogens, a higher rate of infection from *Staphylococcus aureus* was observed in the CASS group (10.1%) compared with the HASS group (1.1%) (*P* < 0.001).

**Table 2:** Distribution of the causative microorganisms in pediatric patients with septic shock

Characteristics	HASS group (*n* = 91)	CASS group (*n* = 207)	*P*
Positive pathogen detection, *n* (%)	55 (60.4)	118 (57.0)	0.580
Positive pathogen detection from aseptic body fluids, *n* (%)	52 (57.1)	96 (46.4)	0.087
Positive pathogen detection from blood, *n* (%)	48 (52.7)	80 (38.6)	0.024
Bacterial culture, *n* (%)			
Gram-positive bacteria	4 (4.4)	64 (30.9)	< 0.001
*Staphylococcus aureus*	1 (1.1)	21 (10.1)	0.006
*Streptococcus pneumoniae*	0 (0.0)	11 (5.3)	0.056
*Staphylococcus epidermidis*	1 (1.1)	8 (3.9)	0.359
*Enterococcus faecium*	2 (2.2)	6 (2.9)	> 0.999
*Streptococcus agalactiae*	0 (0.0)	7 (3.4)	0.174
Group A *Streptococcus*	0 (0.0)	6 (2.9)	0.233
Other Gram-positive bacteria^a^	0 (0.0)	5 (2.4)	0.315
Gram-negative bacteria	43 (47.3)	35 (16.9)	< 0.001
*Klebsiella pneumoniae*	17 (18.7)	7 (3.4)	< 0.001
*Pseudomonas aeruginosa*	11 (12.1)	11 (5.3)	0.039
*Acinetobacter baumanii*	7 (7.7)	11 (5.3)	0.427
*Escherichia coli*	4 (4.4)	4 (1.9)	0.411
*Haemophilus influenzae*	1 (1.1)	2 (1.0)	> 0.999
*Burkholderia*	1 (1.1)	1 (0.5)	> 0.999
*Stenotrophomonas maltophilia*	1 (1.1)	0 (0.0)	0.672
Other gram-negative bacteria^b^	2 (2.2)	2 (1.0)	0.761
Total typical bacteria	47 (51.6)	98 (47.3)	0.493
Type of resistance distribution, *n* (%)			
MDR	36 (39.6)	58 (28)	0.048
XDR	5 (5.5)	5 (2.4)	0.312
Virological studies			
Influenza A virus	3 (3.3)	11 (5.3)	0.645
Influenza B virus	0 (0.0)	6 (2.9)	0.233
Epstein-Barr virus	0 (1.1)	7 (3.4)	0.174
Cytomegalovirus	0 (0.0)	2 (2.2)	0.171
Respiratory syncytial virus	1 (1.1)	3 (1.4)	> 0.999
Parainfluenza virus	1 (1.1)	2 (1.0)	> 0.999
Adenoviruses	0 (0.0)	1 (0.5)	> 0.999
Total virus	5 (5.5)	18 (8.7)	0.340
Fungal culture, *n* (%)			
*Candida albicans*	3 (3.3)	5 (2.4)	0.965
*Candida tropicalis*	1 (1.1)	0 (0.0)	0.672
*Candida parapsilosis*	0 (0.0)	1 (0.5)	> 0.999
*Candida lusitaniae*	1 (1.1)	0 (0.0)	0.672
*Trichosporon asteroides*	1 (1.1)	0 (0.0)	0.672
Total fungus	6 (6.6)	6 (2.9)	0.240
Types of infection, *n* (%)			
Single bacterial infection	47 (51.6)	98 (47.3)	0.493
Multiple bacterial infection	1 (1.1)	4 (1.9)	0.979
Bacterium/virus coinfection	3 (3.3)	6 (2.9)	> 0.999
Bacterium/fungal coinfection	3 (3.3)	6 (2.9)	> 0.999

Classification variables were represented as count (percentage). *HASS* hospital-acquired septic shock, *CASS* community-acquired septic shock, *MDR* multidrug-resistant, *XDR* extensively drug-resistant. ^a^Other Gram-positive bacteria included a strain of *Bacillus cereus*, a strain of *Staphylococcus haemolyticus*, a strain of *Streptococcus bradycariae*, a strain of *Streptococcus parasanguinis*, and a strain of *Mycobacterium*; ^b^other gram-negative bacteria included a strain of *Escherichia vulneris*, a strain of *Neisseria meningitidis* C group, a strain of *Enterobacter cloacae,* and a strain of *Aeromonas hydrophila*

A higher number of patients in the HASS group had Gram-negative bacterial infection (47.3%) compared to the CASS group (16.9%) (*P* < 0.001). The detection rate of *Klebsiella pneumoniae* and *Pseudomonas aeruginosa* in the HASS patients was 18.7% and 12.1%, respectively, higher than those in the CASS group (3.4% and 5.3%; *P* < 0.001 and *P* = 0.039, respectively). A significant difference was observed in MDR bacterial infection between the HASS (39.6%) and CASS groups (28.0%) (*P* = 0.048).

### Supportive and antimicrobial therapies

A significant difference in the use of empirical antimicrobial therapy on the first day between the two groups was observed. The patients in the CASS group were mainly given two antimicrobial drugs (55.1%). In contrast, more than two antimicrobial drugs were used for patients in the HASS group (54.9%) (Table 3).

**Table 3:** Supportive and antimicrobial therapies in pediatric patients with septic shock

Characteristics	HASS group (*n* = 91)	CASS group (*n* = 207)	*P*
Respiratory support, *n* (%)	91 (100.0)	206 (99.5)	> 0.999
Invasive mechanical ventilation	57 (62.6)	143 (69.1)	0.275
Noninvasive ventilation	20 (22.0)	39 (18.8)	0.531
Oxygen therapy	14 (15.4)	24 (11.6)	0.366
Type of antimicrobial drugs at PICU admission, *n* (%)			
One antimicrobial drug only	12 (13.2)	39 (18.8)	0.233
Two antimicrobial drugs	29 (31.9)	114 (55.1)	< 0.001
More than two antimicrobial drugs	50 (54.9)	54 (26.1)	< 0.001
Types of empirical antimicrobial therapy on day 1, *n* (%)			
Carbapenems	61 (67.0)	148 (71.5)	0.438
Glycopeptides	33 (36.3)	75 (36.2)	0.996
Oxazolidinone	35 (38.5)	75 (36.2)	0.715
Beta-lactamase inhibitors	10 (11.0)	27 (13.0)	0.620
Cephalosporin	9 (9.9)	22 (10.6)	0.848
Aminoglycosides	13 (14.3)	4 (1.9)	< 0.001
Quinolones	3 (3.3)	3 (1.4)	0.550
Nitroimidazoles	8 (8.8)	12 (5.8)	0.341
Sulfanilamide	19 (20.9)	4 (1.9)	< 0.001
Glycyl tetracyclines	10 (11.0)	1 (0.5)	< 0.001
Macrolide antibiotics	1 (1.1)	6 (2.9)	0.596
Antiviral drugs, *n* (%)			
Cyclopentanes	5 (5.5)	28 (13.5)	0.042
Neuraminidase inhibitors	0 (0.0)	9 (4.3)	0.098
Nucleoside antiviral drugs	7 (7.7)	10 (4.8)	0.327
Antifungal drugs, *n* (%)			
Polyene antifungal drugs	1 (1.1)	0 (0.0)	0.672
Azole antifungals	26 (28.6)	15 (7.2)	< 0.001
Echinocandin antifungal drugs	16 (17.6)	7 (3.4)	< 0.001
Antimicrobial agent adjustment	21 (23.1)	55 (26.6)	0.524
Glucocorticoids use	26 (28.6)	55 (26.6)	0.721
Vasoactive drugs	76 (83.5)	161 (77.8)	0.258
Renal replacement therapy	17 (18.7)	31 (15.0)	0.423

Classification variables were represented as count (percentage). *HASS* hospital-acquired septic shock, *CASS* community-acquired septic shock, *PICU* pediatric intensive care unit

### Complications and outcome

The CASS group had more patients with cerebral dysfunction compared to the HASS group. No significant differences were observed in the proportion of multiple organ dysfunction syndrome (MODS) and other complications between the two groups. The total 28-day mortality rate of the two groups was 45.0% (134/298). Statistically significant differences were observed between the HASS (62.6%) and CASS groups (37.2%; *P* < 0.001). The in-hospital mortality rate in the HASS group (33.3%) was higher than that of the CASS group (12.1%; *P* < 0.001; Table 4).

**Table 4:** Complications and outcomes in pediatric patients with septic shock

Characteristics	HASS group (*n* = 91)	CASS group (*n* = 207)	*P*
Complications, *n* (%)			
Respiratory failure	76 (83.5)	182 (87.9)	0.304
Acute renal failure	34 (37.4)	76 (36.7)	0.915
Liver dysfunction	28 (30.8)	74 (35.7)	0.404
DIC	28 (30.8)	61 (29.5)	0.821
Cerebral dysfunction	16 (17.6)	61 (29.5)	0.031
MODS	79 (86.8)	179 (86.5)	0.937
Prognosis			
Length of PICU stay, median (IQR)	4 (1, 11)	5 (2, 12)	0.672
Length of hospital stay, median (IQR)	17 (7, 30)	12 (2, 24)	0.004
In-hospital mortality, *n* (%)	30 (33.3)	25 (12.1)	< 0.001
28-d mortality, *n* (%)	57 (62.6)	77 (37.2)	< 0.001

Classification variables were represented as count (percentage). *HASS* hospital-acquired septic shock, *CASS* community-acquired septic shock, *DIC* disseminated intravascular coagulation, *MODS* multiple organ dysfunction syndrome, *IQR* interquartile range, *PICU* pediatric intensive care unit

### Predictive risk factors for 28-day mortality in children with septic shock

In this study the variables mentioned previously (Table 5) were included in the univariable logistic regression analysis of the 28-day mortality rate. For all patients, HASS, combined hematologic/oncologic diseases, PIM2, lactate (LAC), platelet (PLT), activated partial thromboplastin time, international normalized ratio, total bilirubin, mechanical ventilation, vasoactive drug therapy, renal replacement therapy, positive pathogen detection, respiratory failure, renal injury, cerebral dysfunction, and MODS were associated with the 28-day mortality rate. Combined hematologic/oncologic diseases, PLT, invasive mechanical ventilation therapy, MODS, vasoactive drug therapy, and positive pathogen detection were related to the 28-day mortality in the HASS group. In the CASS group, PIM2, activated partial thromboplastin time, international normalized ratio, creatinine, pH value, LAC, invasive mechanical ventilation therapy, respiratory failure, renal injury, disseminated intravascular coagulation, cerebral dysfunction, and renal replacement therapy were associated with the 28-day mortality (Table 5).

**Table 5:** Univariable logistic regression analysis of 28-day mortality in children with septic shock

Variables	Total		HASS group		CASS group	
	*P*	OR (95% CI)	*P*	OR (95% CI)	*P*	OR (95% CI)
HASS	**< 0.001**	2.830 (1.700–4.712)	–	–	–	–
Age	0.689	1.010 (0.963–1.058)	0.790	1.011 (0.931–1.098)	0.524	0.980 (0.921–1.043)
Combined hematologic/oncologic diseases	**< 0.001**	2.939 (1.760–4.908)	**0.015**	3.071 (1.245–7.578)	0.249	1.583 (0.725–3.456)
PIM2	**0.009**	1.016 (1.004–1.028)	0.335	0.989 (0.968–1.011)	**< 0.001**	1.030 (1.014–1.046)
Abnormal HR	0.110	0.688 (0.434–1.089)	0.851	0.921 (0.391–2.171)	0.272	0.726 (0.410–1.286)
Abnormal RR	0.869	0.954 (0.542–1.678)	0.554	1.350 (0.500–3.644)	0.683	0.863 (0.426–1.750)
Abnormal SBP	0.541	0.834 (0.467–1.491)	0.881	0.922 (0.320–2.663)	0.494	0.775 (0.373–1.610)
PH	0.558	1.000 (0.998–1.001)	0.591	0.445 (0.023–8.476)	**< 0.001**	0.019 (0.002–0.151)
BE	0.830	1.004 (0.970–1.039)	0.134	1.045 (0.986–1.107)	0.122	0.964 (0.920–1.010)
LAC	**0.001**	1.102 (1.038–1.170)	0.603	1.029 (0.924–1.146)	**0.001**	1.139 (1.058–1.227)
CRP	0.466	1.001 (0.998–1.004)	0.171	1.004 (0.998–1.009)	0.102	0.996 (0.992–1.001)
PCT	0.328	1.002 (0.998–1.005)	0.301	1.004 (0.997–1.010)	0.322	1.002 (0.998–1.006)
PLT	**< 0.001**	0.997 (0.996–0.999)	**0.010**	0.996 (0.992–0.999)	0.125	0.999 (0.997–1.000)
APTT	**0.007**	1.015 (1.004–1.025)	0.375	1.008 (0.991–1.025)	**0.005**	1.021 (1.006–1.036)
INR	**0.019**	1.673 (1.087–2.575)	0.500	1.354 (0.561–3.270)	**0.016**	1.892 (1.127–3.175)
ALT	0.265	1.000 (1.000–1.000)	0.624	1.000 (0.999–1.001)	0.242	1.000 (1.000–1.001)
TBIL	**0.008**	1.013 (1.003–1.023)	0.062	1.018 (0.999–1.037)	0.593	1.004 (0.990–1.018)
CR	0.205	1.002 (0.999–1.006)	0.740	0.999 (0.992–1.005)	**0.045**	1.005 (1.000–1.009)
Mechanical ventilation	**< 0.001**	5.305 (2.231–12.612)	**0.030**	3.900 (1.143–13.311)	**0.002**	10.548 (2.391–46.531)
Vasoactive drug therapy	**0.016**	2.084 (1.145–3.792)	**0.015**	4.333 (1.335–14.066)	0.283	1.469 (0.727–2.969)
Renal replacement therapy	**0.009**	2.340 (1.238–4.420)	0.455	1.547 (0.493–4.850)	**0.011**	2.746 (1.260–5.984)
Positive pathogen detection	**0.030**	1.679 (1.051–2.683)	**0.004**	3.661 (1.496–8.955)	0.541	1.195 (0.675–2.118)
Respiratory failure	**0.001**	3.818 (1.695–8.603)	0.054	3.060 (0.980–9.553)	**0.006**	8.061 (1.845–35.225)
Renal injury	**0.006**	1.961 (1.217–3.159)	0.100	2.170 (0.861–5.469)	**0.022**	1.976 (1.104–3.537)
DIC	0.129	1.471 (0.894–2.421)	0.471	0.715 (0.288–1.778)	**0.022**	2.038 (1.107–3.753)
Cerebral dysfunction	**0.027**	0.553 (0.328–0.934)	0.579	0.721 (0.227–2.288)	**0.004**	0.404 (0.219–0.745)
MODS	**0.003**	3.237 (1.482–7.071)	**0.003**	11.458 (2.332–56.307)	0.156	1.927 (0.778–4.770)

The letters in bold font indicating significant differences. *HASS* hospital-acquired septic shock, *CASS* community-acquired septic shock, *PIM2* pediatric index of mortality 2, *HR* heart rate, *RR* respiratory rate, *SBP* systolic blood pressure, *BE* base excess, *LAC* lactate, *CRP* C-reactive protein, *PCT* procalcitonin, *PLT* platelet, *APTT* activated partial thromboplastin time, *INR* international normalized ratio, *ALT* alanine aminotransferase, *TBIL* total bilirubin, *CR* creatinine, *DIC* disseminated intravascular coagulation, *MODS* multiple organ dysfunction syndrome, *CI* confidence interval, *OR* odds ratio

The results of multivariable logistic regression analysis after excluding the collinear predictors showed that HASS, combined hematologic/oncologic disease, LAC, PLT, mechanical ventilation, positive pathogen detection, renal injury, and MODS were risk factors in all children with septic shock. PLT, positive pathogen detection and MODS were risk factors for 28-day mortality in the HASS group. LAC and mechanical ventilation were risk factors for 28-day mortality in the CASS group (Table 6).

**Table 6:** Predictors of 28-day mortality in children with septic shock from multivariable logistic regression analysis

Variables	All		HASS group		CASS group	
	*P*	OR (95% CI)	*P*	OR (95% CI)	*P*	OR (95% CI)
HASS	**0.015**	2.264 (1.170–4.382)	–	–	–	–
Combined hematologic/oncologic diseases	**0.008**	2.476 (1.265–4.846)	–	–	–	–
LAC	**0.017**	1.083 (1.015–1.155)	–	–	**0.012**	1.104 (1.022–1.192)
PLT	–	–	**0.028**	0.996 (0.992–1.000)	–	–
Mechanical ventilation	**0.001**	5.528 (2.095–14.582)	–	–	**0.006**	8.114 (1.806–36.465)
Positive pathogen detection	**0.026**	1.861 (1.079–3.211)	**0.013**	3.557 (1.307–9.684)	–	–
Renal injury	**0.038**	2.119 (1.044–4.302)	–	–	–	–
MODS	–	–	**0.006**	10.953 (1.974–60.775)	–	–

The letters in bold font indicating significant differences. APTT and INR were colinear. APTT was not included in the model. *HASS* hospital-acquired septic shock, *CASS* community-acquired septic shock, *LAC* lactate, *PLT* platelet, *MODS* multiple organ dysfunction syndrome, *APTT* activated partial thromboplastin time, *INR* international normalized ratio, *CI* confidence interval, *OR* odds ratio

## Discussion

In the present study several differences were observed between pediatric patients with HASS and CASS. The underlying diseases, pathogens, infection sites, and mortality varied widely between HASS and CASS; in addtion, the predictors of 28-day mortality were different.

Several studies have been conducted to assess the differences between HASS and CASS, but the study on Chinese children is limited. Regarding the difference in basic diseases, published data have suggested that most blood malignancies in HASS patients can lead to immune deficiency, which is a risk factor for infection and death [19–21]. In addition, neutropenia was shown to be one of the factors that could increase the risk of infection [22]. HASS patients presented bloodstream and digestive tract infections, which was similar to the findings by Westphal et al. and Baker et al. [23, 24]. Baker et al. demonstrated that chemotherapy damages the gastrointestinal mucosa and that enterogenic sepsis and bacteremia occur in hospitals [24]. The main site of CASS was the respiratory tract, which was in line with the results of the current study [23]. Significant differences have previously been observed in microbiological infection profiles between HASS and CASS patients [25, 26]. Under the influence of gastrointestinal mucositis caused by chemotherapy and long-term neutropenia, children with nosocomial infections, especially hematologic neoplasms, were at high risk of Gram-negative bacteremia [24]. Nonetheless, the predominant type of pathogen in CASS is Gram-positive bacteria [27].

The 28-day mortality of patients varied in the HASS and CASS groups. Similar conclusions were reached for a multicenter cohort study, in which the mortality rates from nosocomial and community-acquired infections were 64.6% and 37.5%, respectively [28]. A previous study showed that immune deficiency and granulocytopenia increased the risk of death from sepsis, and the majority of children in our study were hospitalized for chemotherapy due to hematologic neoplasms diseases [29].

The predictors of 28-day mortality in the two groups varied remarkably. The multivariable logistic regression analysis showed that the risk of 28-day mortality in HASS increased with positive pathogen detection or MODS. PLT was shown to be a protective factor for mortality in children with HASS. In patients with CASS, the increase of LAC and the need for mechanical ventilation were risk factors for 28-day mortality. Another study found that bloodstream infections acquired in the intensive care unit were related to increased mortality [30], which was consistent with the present study. Furthermore, MODS might be a predictor of sepsis mortality, which has been verified in previous studies [31–33]. On the other hand, some studies have analyzed MODS as a dependent variable for outcome or prognosis [34]. In this study organ function was restored in 60.9% of patients with CASS post-treatment. In the HASS group, 30.4% of children with MODS survived; therefore, irreversible MODS is deemed a direct risk factor for death from septic shock because MODS is a severe manifestation [35]. Several studies revealed that PLT should be included in the prognosis model of sepsis in children [36, 37]. The prognostic power of PLT has been studied extensively in adults [38–40], and PLT might play a key role in coordinating the host inflammatory response [38]. Hematological/oncological diseases were predominant in the HASS group. Due to tumor and chemotherapy, white blood cell and PLT levels were lower than normal, and the effect on prognosis was more prominent than in patients with normal PLT count and function. Therefore, the predictive value of PLT should be explored according to the characteristics of different populations. Accumulating evidence from adults and children showed that LAC was related to the prognosis of septic shock [41–44], and most of the cases had community-acquired infections, which was consistent with the present study. Similar to previous studies, the mortality in the CASS group could be predicted by mechanical ventilation [37, 45]. Due to severe conditions of patients treated with invasive ventilators and to adverse effects of invasive ventilation on hemodynamics, the need for invasive mechanical ventilation is a risk factor for mortality [46].

The highlights of the current findings were as follows: first, the children in the HASS group had significantly fewer complaints than those in the CASS group. Similar findings were noted in the study by Heinz et al. [47], wherein the patients with neoplasm of neutropenia presented atypical or indistinct signs and symptoms of infection, and fever might be the sole clinical symptom. Therefore, early attention should be paid to fever in HASS pediatric patients. Second, many Gram-negative bacterial infections in CASS were involved in nosocomial infections. It is not uncommon for these drug-resistant Gram-negative bacteria to cause infection in the community and have drug resistance [48–51] because the patients often have underlying diseases [52]. In a study in Milan conducted on adults, the resistance rate of the community infection was about 17% [53]. In a pediatric study in China, the overall resistance rate of Gram-negative bloodstream infections was 60.1% [54]. The high drug resistance rate of community infection requires the attention of medical staff and society. The emergence of drug-resistant bacteria poses significant challenges to the selection of antibiotics and the risk of death. Third, in this study the first antibiotic selection for CASS patients was excessive. Although the antibiotics were adjusted after obtaining the microbial results, the selection should be based on guidelines, patient status, local microorganisms, and drug resistance. Fourth, PIM2 is a predictor of death but not HASS in the present study. Interestingly, PIM2 estimates were significantly lower than the actual 28-day mortality rate. Several studies confirmed that PIM2 has a good prediction ability for critical illness but differed significantly from that in the HASS group. Edwards et al. and Fonseca et al. revealed a higher mortality rate for complex chronic conditions than predicted by PIM2 [55, 56]. In a report in 2014, PIM2 was shown to have a significant effect on treatment prior to admission to the PICU, and both age-uncorrected physical sign data and the inapplicability of high-risk diagnoses of PIM2-specific regions may be biased [57]. In addition to the above factors, unscheduled hospitalization also increased the bias between PIM2 predictions and actual deaths in our data.

Nevertheless, the current study had some limitations. First, this was a single-center retrospective cohort study that relied on routine clinical data, with lost cases and a small amount of missing data on the variables. Second, although the total number of cases was approximately 300, data of multifactor regression analysis after grouping were less. Because some patients were transferred to our hospital after they developed septic shock, information on neutrophils, anemia, PLT, central venous catheterization, immunosuppressive state, mucosal barrier destruction, and other potential organ dysfunctions could not be obtained. Third, we found that the mortality of children with septic shock was extremely high, that the high-grade antibiotics were the preferred antimicrobial drugs, and that high rates of drug-resistant bacteria were detected in the CASS group. However, because the study was based on a special population and treatment measures, the conclusions cannot be applied to other PICUs indiscriminately. For the logistic regression model, we required more patients to ensure the stability of the model; therefore, more outcome events are imperative to substantiate these findings.

In conclusion, the underlying diseases, pathogens, complications, prognosis, and mortality rates varied widely between HASS and CASS. The predictors of 28-day mortality differed between HASS and CASS pediatric patients with septic shock.

## Data Availability

The data from this study are available from the corresponding author upon reasonable request.

## References

[1] Weiss SL, Peters MJ, Alhazzani W, Agus MSD, Flori HR, Inwald DP (2020). Surviving sepsis campaign international guidelines for the management of septic shock and sepsis-associated organ dysfunction in children. Intensive Care Med.

[2] Wang Y, Sun B, Yue H, Lin X, Li B, Yang X (2014). An epidemiologic survey of pediatric sepsis in regional hospitals in China. Pediatr Crit Care Med.

[3] Fleischmann-Struzek C, Goldfarb DM, Schlattmann P, Schlapbach LJ, Reinhart K, Kissoon N (2018). The global burden of paediatric and neonatal sepsis: a systematic review. Lancet Respir Med.

[4] Weiss SL, Fitzgerald JC, Pappachan J, Wheeler D, Jaramillo-Bustamante JC, Salloo A (2015). Global epidemiology of pediatric severe sepsis: the sepsis prevalence, outcomes, and therapies study. Am J Respir Crit Care Med.

[5] Balamuth F, Weiss SL, Neuman MI, Scott H, Brady PW, Paul R (2014). Pediatric severe sepsis in U.S. children's hospitals. Pediatr Crit Care Med..

[6] Tan B, Wong JJ, Sultana R, Koh JCJW, Jit M, Mok YH (2019). Global case-fatality rates in pediatric severe sepsis and septic shock: a systematic review and meta-analysis. JAMA Pediatr.

[7] Singer M, Deutschman CS, Seymour CW, Shankar-Hari M, Annane D, Bauer M (2016). The third international consensus definitions for sepsis and septic shock (Sepsis-3). JAMA.

[8] Briassoulis P, Briassoulis G, Christakou E, Machaira M, Kassimis A, Barbaressou C (2021). Active surveillance of healthcare-associated infections in pediatric intensive care units: multicenter ECDC HAI-net ICU protocol (v2.2) implementation, antimicrobial resistance and challenges. Pediatr Infect Dis J..

[9] Boeddha NP, Schlapbach LJ, Driessen GJ, Herberg JA, Rivero-Calle I, Cebey-López M (2018). Mortality and morbidity in community-acquired sepsis in European pediatric intensive care units: a prospective cohort study from the European Childhood Life-threatening Infectious Disease Study (EUCLIDS). Crit Care.

[10] Murni IK, Duke T, Daley AJ, Kinney S, Soenarto Y (2019). Predictors of mortality in children with nosocomial bloodstream infection. Paediatr Int Child Health.

[11] Vila Pérez D, Jordan I, Esteban E, García-Soler P, Murga V, Bonil V (2014). Prognostic factors in pediatric sepsis study, from the Spanish Society of Pediatric Intensive Care. Pediatr Infect Dis J.

[12] Giannoni E, Agyeman PKA, Stocker M, Posfay-Barbe KM, Heininger U, Spycher BD (2018). Neonatal sepsis of early onset, and hospital-acquired and community-acquired late onset: a prospective population-based cohort study. J Pediatr.

[13] Tian HC, Zhou JF, Weng L, Hu XY, Peng JM, Wang CY (2019). Epidemiology of Sepsis-3 in a sub-district of Beijing: secondary analysis of a population-based database. Chin Med J (Engl).

[14] Subspecialty Group of Emergency Medicine, the Society of Pediatrics, Chinese Medical Association; Subspecialty Group of Pediatrics, the Society of Emergency Medicine, Chinese Medical Association; Pediatric Emergency Medicine Physicians, Chinese Medical Doctor Association. Expert consensus for the diagnosis and management of septic shock (infectious shock) in children (2015). Zhonghua Er Ke Za Zhi. 2015;53:576–80 **(in Chinese)**. 26717653

[15] Friedman ND, Kaye KS, Stout JE, McGarry SA, Trivette SL, Briggs JP (2002). Health care-associated bloodstream infections in adults: a reason to change the accepted definition of community-acquired infections. Ann Intern Med.

[16] Magiorakos AP, Srinivasan A, Carey RB, Carmeli Y, Falagas ME, Giske CG (2012). Multidrug-resistant, extensively drug-resistant and pandrug-resistant bacteria: an international expert proposal for interim standard definitions for acquired resistance. Clin Microbiol Infect.

[17] Goldstein B, Giroir B, Randolph A, International Consensus Conference on Pediatric Sepsis. International pediatric sepsis consensus conference: definitions for sepsis and organ dysfunction in pediatrics. Pediatr Crit Care Med. 2005;6:2–8. 10.1097/01.PCC.0000149131.72248.E6 15636651

[18] Ramírez M (2013). Multiple organ dysfunction syndrome. Curr Probl Pediatr Adolesc Health Care.

[19] Hall A, Lynagh M, Tzelepis F, Paul C, Bryant J (2016). How can we help haematological cancer survivors cope with the changes they experience as a result of their cancer?. Ann Hematol.

[20] Battaglia CC, Hale K (2019). Hospital-acquired infections in critically ill patients with cancer. J Intensive Care Med.

[21] Montassier E, Batard E, Gastinne T, Potel G, de La Cochetière MF (2013). Recent changes in bacteremia in patients with cancer: a systematic review of epidemiology and antibiotic resistance. Eur J Clin Microbiol Infect Dis.

[22] Pana ZD, Roilides E, Warris A, Groll AH, Zaoutis T (2017). Epidemiology of invasive fungal disease in children. J Pediatric Infect Dis Soc.

[23] Westphal GA, Pereira AB, Fachin SM, Barreto ACC, Bornschein ACGJ, Caldeira Filho M, et al. Characteristics and outcomes of patients with community-acquired and hospital-acquired sepsis. Rev Bras Ter Intensiva. 2019;31:71–8 **(in Portuguese, English)**. 10.5935/0103-507X.20190013 PMC6443308 30970093

[24] Baker TM, Satlin MJ (2016). The growing threat of multidrug-resistant Gram-negative infections in patients with hematologic malignancies. Leuk Lymphoma.

[25] Matta R, Hallit S, Hallit R, Bawab W, Rogues AM, Salameh P (2018). Epidemiology and microbiological profile comparison between community and hospital acquired infections: a multicenter retrospective study in Lebanon. J Infect Public Health.

[26] Cardoso T, Ribeiro O, Aragão I, Costa-Pereira A, Sarmento A (2013). Differences in microbiological profile between community-acquired, healthcare-associated and hospital-acquired infections. Acta Med Port.

[27] Droz N, Hsia Y, Ellis S, Dramowski A, Sharland M, Basmaci R (2019). Bacterial pathogens and resistance causing community acquired paediatric bloodstream infections in low- and middle-income countries: a systematic review and meta-analysis. Antimicrob Resist Infect Control.

[28] Dabar G, Harmouche C, Salameh P, Jaber BL, Jamaleddine G, Waked M (2015). Community- and healthcare-associated infections in critically ill patients: a multicenter cohort study. Int J Infect Dis.

[29] Tolsma V, Schwebel C, Azoulay E, Darmon M, Souweine B, Vesin A (2014). Sepsis severe or septic shock: outcome according to immune status and immunodeficiency profile. Chest.

[30] Adrie C, Garrouste-Orgeas M, Ibn Essaied W, Schwebel C, Darmon M, Mourvillier B (2017). Attributable mortality of ICU-acquired bloodstream infections: Impact of the source, causative micro-organism, resistance profile and antimicrobial therapy. J Infect.

[31] Shah S, Deshmukh CT, Tullu MS (2020). The predictors of outcome and progression of pediatric sepsis and septic shock: a prospective observational study from western India. J Postgrad Med.

[32] Kutko MC, Calarco MP, Flaherty MB, Helmrich RF, Ushay HM, Pon S (2003). Mortality rates in pediatric septic shock with and without multiple organ system failure. Pediatr Crit Care Med.

[33] Lalitha AV, Satish JK, Reddy M, Ghosh S, George J, Pujari C (2021). Sequential organ failure assessment score as a predictor of outcome in sepsis in pediatric intensive care unit. J Pediatr Intensive Care.

[34] Moustafa AA, Antonios MA, Abdellatif EM, Hussain AH (2018). Association of lactate/albumin ratio level to organ failure and mortality in severe sepsis in a pediatric intensive care unit in Egypt. Turk J Pediatr.

[35] Weiss SL, Balamuth F, Hensley J, Fitzgerald JC, Bush J, Nadkarni VM (2017). The epidemiology of hospital death following pediatric severe sepsis: when, why, and how children with sepsis die. Pediatr Crit Care Med.

[36] Wong HR, Cvijanovich NZ, Anas N, Allen GL, Thomas NJ, Bigham MT (2016). Pediatric sepsis biomarker risk model-II: redefining the pediatric sepsis biomarker risk model with septic shock phenotype. Crit Care Med.

[37] Menon K, Schlapbach LJ, Akech S, Argent A, Biban P, Carrol ED (2022). Criteria for pediatric sepsis—a systematic review and meta-analysis by the pediatric sepsis definition taskforce. Crit Care Med.

[38] Al Harbi G, Chaari A (2020). Platelets parameters in septic shock: clinical usefulness and prognostic value. Blood Coagul Fibrinolysis.

[39] Kim JH, Lee Y, Cho YS, Sohn YJ, Hyun JH, Ahn SM (2021). A modified simple scoring system using the red blood cell distribution width, delta neutrophil index, and mean platelet volume-to-platelet count to predict 28-day mortality in patients with sepsis. J Intensive Care Med.

[40] Menard CE, Kumar A, Houston DS, Turgeon AF, Rimmer E, Houston BL (2019). Evolution and impact of thrombocytopenia in septic shock: a retrospective cohort study. Crit Care Med.

[41] Ryoo SM, Lee J, Lee YS, Lee JH, Lim KS, Huh JW (2018). Lactate level versus lactate clearance for predicting mortality in patients with septic shock defined by Sepsis-3. Crit Care Med.

[42] Schlapbach LJ, MacLaren G, Festa M, Alexander J, Erickson S, Beca J (2017). Prediction of pediatric sepsis mortality within 1 h of intensive care admission. Intensive Care Med.

[43] Choi SJ, Ha EJ, Jhang WK, Park SJ (2021). Association between the lactate/albumin ratio and mortality in pediatric septic shock patients with underlying chronic disease: retrospective pilot study. Minerva Pediatr (Torino).

[44] Alam A, Gupta S (2021). Lactate measurements and their association with mortality in pediatric severe sepsis in India: evidence that 6-hour level performs best. J Intensive Care Med.

[45] Chen M, Lu X, Hu L, Liu P, Zhao W, Yan H (2017). Development and validation of a mortality risk model for pediatric sepsis. Medicine (Baltimore).

[46] de Montmollin E, Aboab J, Ferrer R, Azoulay E, Annane D (2016). Criteria for initiation of invasive ventilation in septic shock: an international survey. J Crit Care.

[47] Heinz WJ, Buchheidt D, Christopeit M, von Lilienfeld-Toal M, Cornely OA, Einsele H (2017). Diagnosis and empirical treatment of fever of unknown origin (FUO) in adult neutropenic patients: guidelines of the Infectious Diseases Working Party (AGIHO) of the German Society of Hematology and Medical Oncology (DGHO). Ann Hematol.

[48] Restrepo MI, Babu BL, Reyes LF, Chalmers JD, Soni NJ, Sibila O (2018). Burden and risk factors for *Pseudomonas aeruginosa* community-acquired pneumonia: a multinational point prevalence study of hospitalised patients. Eur Respir J.

[49] McCarthy KL, Paterson DL (2017). Community-acquired *Pseudomonas aeruginosa* bloodstream infection: a classification that should not falsely reassure the clinician. Eur J Clin Microbiol Infect Dis.

[50] Meumann EM, Anstey NM, Currie BJ, Piera KA, Kenyon JJ, Hall RM (2019). Genomic epidemiology of severe community-onset *Acinetobacter baumannii* infection. Microb Genom.

[51] Chen CT, Wang YC, Kuo SC, Shih FH, Chen TL, How CK (2018). Community-acquired bloodstream infections caused by *Acinetobacter baumannii*: a matched case-control study. J Microbiol Immunol Infect.

[52] Yo CH, Hsein YC, Wu YL, Hsu WT, Ma MH, Tsai CH (2019). Clinical predictors and outcome impact of community-onset polymicrobial bloodstream infection. Int J Antimicrob Agents.

[53] Capsoni N, Bellone P, Aliberti S, Sotgiu G, Pavanello D, Visintin B (2019). Prevalence, risk factors and outcomes of patients coming from the community with sepsis due to multidrug resistant bacteria. Multidiscip Respir Med.

[54] Dong L, Zhang XY, Li CC, Li Z, Xia YQ (2017). Characteristics of epidemiology and antimicrobial resistance of gram-negative bacterial bloodstream infections in children. Zhonghua Er Ke Za Zhi.

[55] Edwards JD, Houtrow AJ, Vasilevskis EE, Rehm RS, Markovitz BP, Graham RJ, et al. Chronic conditions among children admitted to U.S. pediatric intensive care units: their prevalence and impact on risk for mortality and prolonged length of stay*. Crit Care Med. 2012;40:2196–203. 10.1097/CCM.0b013e31824e68cf PMC3378726 22564961

[56] Fonseca JG, Ferreira AR (2014). Application of the pediatric index of mortality 2 in pediatric patients with complex chronic conditions. J Pediatr (Rio J).

[57] Shukla VV, Nimbalkar SM, Phatak AG, Ganjiwale JD (2014). Critical analysis of PIM2 score applicability in a tertiary care PICU in Western India. Int J Pediatr.

